# Effects of a therapeutic suit based on myofascial meridians on postural control and balance in children with cerebral palsy: a multiple-baseline, single-subject study

**DOI:** 10.3389/fped.2025.1459839

**Published:** 2025-01-20

**Authors:** Thalita Karla Flores Cruz, Deisiane Oliveira Souto, Rafhael Guimarães Capuchinho, Arthur Felipe Barroso de Lima, Amanda Aparecida Alves Cunha Nascimento, Ianara Pereira Silva, Patrícia Aparecida Neves Santana, Lia Constantino Criscoullo, Vitor Geraldi Haase

**Affiliations:** ^1^ Postgraduate Program in Neurosciences, Universidade Federal de Minas Gerais, Belo Horizonte, Brazil; ^2^ Postgraduate Program in Rehabilitation Sciences, Universidade Federal de Minas Gerais, Belo Horizonte, Brazil; ^3^ Reabilitar Rehabilitation Clinic, Ribeirão das Neves, Minas Gerais, Brazil; ^4^ Postgraduate Program in Psychology: Cognition and Behavior, Universidade Federal de Minas Gerais, Belo Horizonte, Brazil

**Keywords:** cerebral palsy, balance, postural control, therapeutic suit, TREINI Exoflex

## Abstract

**Aim:**

To investigate the effects of an intervention programme based on the TREINI Exoflex therapeutic suit on balance, postural control, activity, and participation outcomes in children with cerebral palsy (CP).

**Method:**

A multiple-baseline, single-subject A/B research design was used. Balance, postural control, mobility, activities of daily living (ADLs), and goal attainment measures were collected for four children with CP. The intervention was a programme designed for the use of a therapeutic suit, that is, the TREINI Exoflex. The 2-SD band and percentage of non-overlapping data methods were used to compare outcomes between the baseline and intervention phases.

**Results:**

The intervention improved balance and postural control in all four children. The scope of the intervention on activity and participation outcomes varied between children. All children showed improvements in at least one stipulated goal and two in mobility and ADLs. Improvements in goal achievement occurred mainly for balance-related goals, whereas behavioural goals were not achieved.

**Conclusion:**

The results of this study support the use of the TREINI Exoflex suit during functional activities by children with CP. Future research should examine the effects of this approach in children of different ages and at different functional levels.

## 1 Introduction

Postural control and balance constitute crucial components for the safe performance of motor tasks and activities of daily living (ADLs). Furthermore, the development of motor skills and coordination occurs because of interactions in the context of performing a specific task (1). If postural control and balance are compromised, environmental interaction may be limited. Postural control is often a challenge for children with cerebral palsy (CP) (2). Difficulties with postural control in children with CP occur from early childhood, even after they have acquired the ability to stand and walk independently (2). Children present with deficiencies in both static and dynamic situations (2). Even during periods of static positioning, they demonstrate greater oscillation of the centre of pressure, as well as the speed of centre of pressure displacement, compared to their typically developing peers (2). This suggests less precise and less efficient postural control in children with CP.

Children with CP also present with sensory impairments, including proprioceptive impairments, that could contribute to impaired postural and balance control (3). Proprioceptive information is an important regulatory mechanism of postural control and balance. Many studies about the innervation of fascial tissue demonstrated its role in proprioception, with neuromuscular spindles and Golgi tendon organs observed deep in the fascial tissue (4). The fascial tissue, a dense irregular connective tissue, surrounds and connects every muscle, organ, and structure in the body, forming continuity throughout the body and playing a crucial role in posture and movement and providing an integrated functional structure (5, 6). Also, this tissue is populated by a dense network of mechanoreceptors, so the fascial tissue can also perform proprioceptive functions (5, 7).

The CP muscle differs structurally, biologically, and mechanically from those of typically developing children of the same age (8). The primary function of skeletal muscles is to produce force and movement, and there is substantial evidence that spastic muscles in children with CP are reduced in these functions compared to typical development (8). Factors that can affect muscle stiffness include changes in the intrinsic mechanical properties of myofibers (i.e., myofiber stiffness), (2) intramuscular connective tissue, or (3) altered fascial loads from the spastic muscle's epimuscular fascial connections with extramuscular connective tissues, synergists, and/or antagonist muscles (9). Muscle hypertonia in individuals with CP is also explained by intrinsic changes in the muscle's pathomorphology — including abnormalities in sarcomerogenesis, decreased number and function of satellite cells, increased connective tissue and fat, highly elastic myofibrils, and ribosomal dysfunction — suggesting a more localized role in contracture development (10–12). Impaired muscle growth, incompatible with more normal increases in bone length, along with significant increases in intramuscular connective tissue, appear to be more primary determinants of static contracture development in CP (10–12). Thus, changes in fascial tissue represent significant contributing factors to force production and movement generation in children with CP. Because changes in fascial tissue directly affect the function of muscle spindles, alterations in its properties impact the quality of proprioceptive information sent to the central nervous system (13).

Based on the premise that the muscles of the human body do not function as independent units, but as a part of an interconnected network throughout the body, with fascial structures acting as linking components, therapies targeting fascial tissue are guided by concepts of myofascial meridians. The myofascial meridians consist of the anatomical connections among fascia, muscles and bone exhibiting mechanical properties consistent with tensegrity structures (13–15). Tensegrity structures describe a principle of structural relationship in which the shape or function is ensured by the closed tensional behaviors of a system, maintaining its equilibrium (16). According to that, Myers (16) defined 11 myofascial meridians connecting distant parts of the body through muscles and fascial tissues. The central rule for selecting the components of a meridian is a direct linear connection between two muscles, provided by the fascial tissue (16).

**Figure 1:**
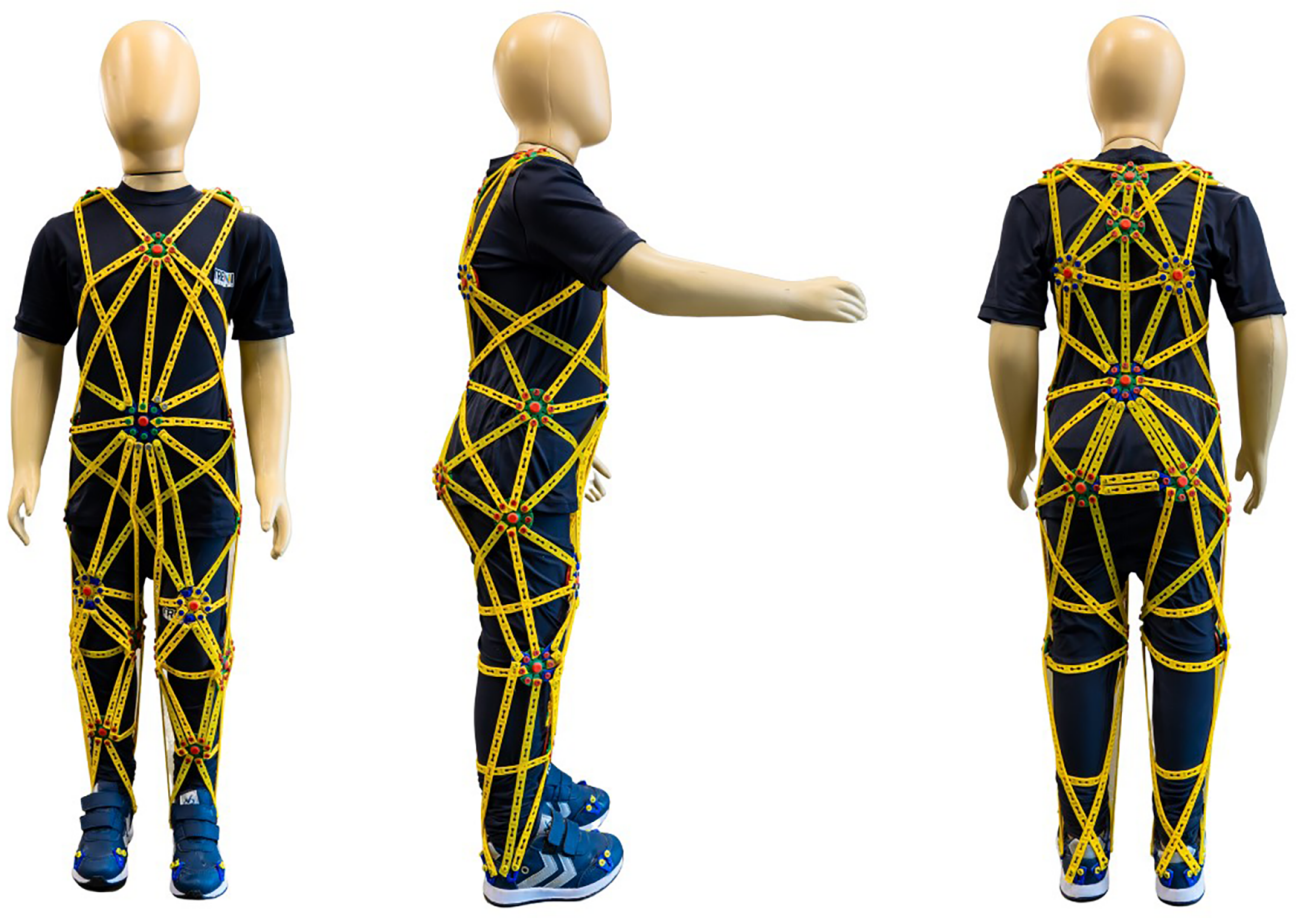
The TREINI exoflex suit (based on the myofascial meridians).

Because of the relevance of improving postural control and balance in children with CP, and the role of fascial tissue innervation in proprioception, a group of researchers developed a therapeutic suit—the TREINI Exoflex (Figure 1). The TREINI Exoflex suit is based on myofascial meridians to improve postural stability and balance, as well as enhancing the individual's proprioceptive information and muscle performance. The TREINI Exoflex operates using the principles of myofascial meridians to enhance tension transmission and facilitate the implementation of complex motor synergies (16, 17). This innovative suit acts as a mediator between the musculoskeletal system and the central nervous system, improving the conduction of sensory information (4, 13). Its structure is designed to provide support without restricting movement, promoting core stability, transmitting force through fascia, and facilitating postural corrections in children (4, 17). The mechanism of action of TREINI Exoflex is optimized when used in conjunction with functional tasks, enhancing therapeutic benefits and promoting a holistic approach. In naturalistic settings, such as the “City of Tomorrow”, the suit improves postural stabilization, optimizes functional postures, and encourages active movements. Additionally, it contributes to increasing muscle strength by transmitting force and resisting muscle contraction through its viscoelastic strips. The effects of the suit are achieved through the strategic distribution of stimulation along the superficial myofascial meridians (anterior and posterior lines) and the lateral and spiral functional lines, stimulating long kinetic chains instead of isolated muscle groups. This approach can generate significant mechanical and proprioceptive benefits, combining stability, postural compensation, and active movement with increased muscle strength (4, 13, 17).

This study employed an A/B research design (SSRD) with a single subject and multiple baselines to primarily evaluate the efficacy of the TREINI Exoflex vest in enhancing postural control and balance in a specific intervention program. Multiple baselines can also allow for the recording of different types of changes seen as the intervention progresses. Given that the timing of intervention introduction is different for each individual, the case for causal relationships is strengthened when changes occur consistently with the introduction of the intervention, regardless of when it was introduced or the experimental conditions under which it was introduced.

Our main hypothesis is that the intervention with the TREINI Exoflex will result in significant improvements in the postural control and balance of children with CP. As a secondary objective, we investigated the program's efficacy in the activity and participation of these children, hypothesizing that the intervention will also promote improvements in these outcomes.

## 2 Method

### 2.1 Study design

A multiple-baseline, single subject A–B research design (MBD SSRD) and random assignment study was conducted (8). An MBD SSRD provides a higher level of evidence than a simple A–B SSRD or a withdrawal A–B–A SSRD (18). This design was chosen to increase the rigor and level of evidence provided by the SSRD, in line with current literature (18–21). Additionally, this design is classified as Level I of evidence by the ranking SSRD described from the American Academy for Cerebral Palsy and Developmental Medicine - AACPDM Methodology for Systematic Reviews (19). The results of these studies are reported in accordance with the Single Case Reporting in Behavioral Interventions (SCRIBE) Guidelines (22).

In a single-subject design, each participant serves as their own control, which involves multiple, repeated observations. The baseline (phase A) measurements were recorded for three and five moments, with random assignment of individuals to length of baseline (phase A), followed by a 6-week intervention (phase B). In this study, case randomization was adopted, wherein cases are randomly assigned to levels N of the design, as described by Levin and Ferron (23). During the intervention phase (phase B), all children were assessed once a week. All children continued to perform routine activities, attending routine therapy sessions (physical therapy and/or occupational therapy once a week) and regular school. Children's therapists were informed about participation in the study and maintained the treatment plans during the study period.

### 2.2 Participants

Children classified in levels I, II, or III of the Gross Motor Function Classification System (GMFCS), who had not undergone orthopedic surgery or received botulinum neurotoxin A in the last 6 months were included. Four children with a mean age of 11 years 9 months (SD = 2 years 6 months) participated in the study. General information about the participants and their caregivers is presented in Table 1. Guardians signed an informed consent form for their children, and children gave oral assent to participate in the study, which was approved by the research ethics committee (no. CAAE-70890923.0.0000.5134).

**Table 1:** Participants characteristics.

	C1	C2	C3	C4
Participants				
Sex	M	M	F	F
Type	Bilateral spastic	Bilateral spastic	Unilateral spastic	Bilateral spastic
Age	14	9	12	11
Height (m)	1.42	1.23	1.57	1.37
Weight (kg)	31.9	24	52	35
GMFCS	II	III	I	III
RCPM	III	IV	IV	IV
Use of orthoses	No	AFO	No	AFO
Schooling	Incomplete elementary school	Incomplete elementary school	Incomplete elementary school	Incomplete elementary school
Caregiver				
Caregiver education	Complete high school	University graduation	Complete high school	University graduation
Caregiver occupation	Maid	History teacher	Hairdresser	Clothing salesperson

Legend: M, male; F, female; C1, child 1; C2, child 2; C3, child 3; C4, child 5; GMFCS, gross motor function classification system; RCPM, Raven's coloured progressive matrices; AFO, ankle–foot orthosis.

### 2.3 Primary outcome measures

In order to assess balance, anticipatory postural control, and functional mobility we used the Timed Up and Go (TUG) (24). During its application, the child is asked to get up from the chair, walk 3 meters, turn around, return to the chair, and sit down again. The time taken to complete the route is recorded. Its validity and reliability have been documented in ambulatory children with CP (25).

In order to assess functional balance ability, Pediatric Balance Scale (PBS) was used. PBS was developed to examine static and dynamic balance; it consists of 14 items that evaluate the functional activities a child can perform during daily life. The PBS has been adapted for the Brazilian population (26); its measurement properties have been evaluated and have shown good responsiveness (27).

### 2.4 Secondary outcome measures

In order to assess the magnitude of body oscillation along the three axes, we used Baiobit (BTS Bioengineering, Milan, Italy) device. In this study, analyses of the magnitude of body sway with eyes open and eyes closed were included. The Baiobit software produces a report that includes a centre of mass (COM) statokinesiogram, as well as results related to the ellipse area, amplitude and displacement, and the length of the trajectory of COM (28).

In order to assess activity (performance), the Pediatric Evaluation of Disability Inventory-Computer Adaptive Test (PEDI-CAT) was used. It is a parent-reported measure of function for children and youth up to 21 years of age. PEDI-CAT uses a computer adaptive platform with an item bank of 276 items. In this study, we analyzed data from the “daily activities” and “mobility” domains. Each test was scored using a standard metric so that results could be compared across time points and groups of children. PEDI-CAT has strong construct validity and reliability in children with CP (29).

In order to assess the participation of children with CP, the Canadian Occupational Performance Measure (COPM) was used. It is a semi-structured interview during which parents list treatment priorities for their children and rate performance and satisfaction scores for those priorities on a scale of 1–10. In addition to being a reliable and validated instrument, the COPM can detect changes in performance and satisfaction over time and after the intervention, thus providing good responsiveness (30).

### 2.5 The intervention program (phase B)

**Figure 2:**
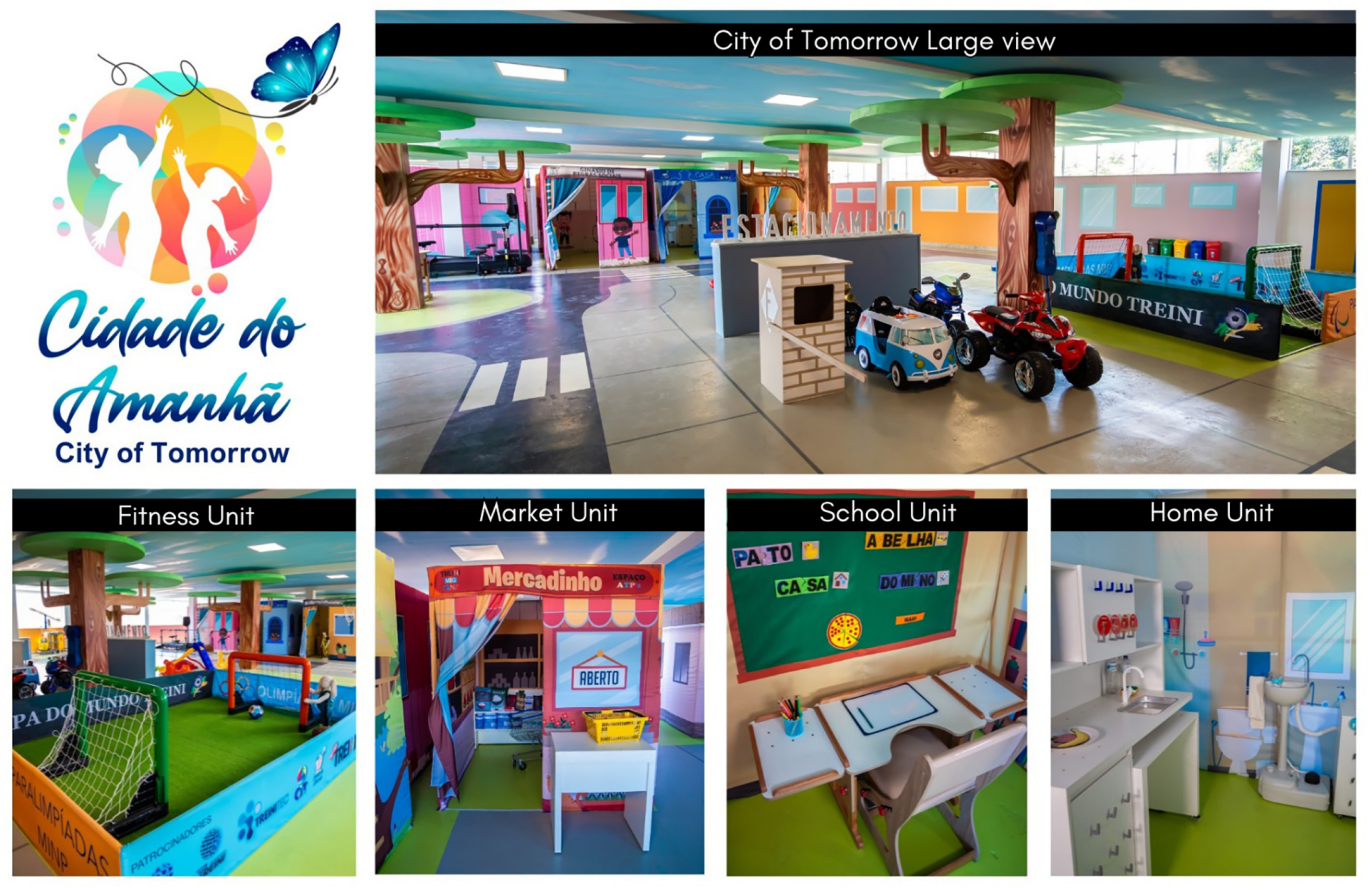
Naturalistic therapeutic environment: the city of tomorrow.

The intervention protocol was used for 6 weeks, 90 min a day, three times a week. The primary element of the intervention program was the TREINI Exoflex, a therapeutic suit developed. This is a provisional file, not the final typeset article from lightweight, thermo-adjustable, and comfortable fabric (84% polyamide, 16% elastane) and interconnected viscoelastic strips (consisting of a non-toxic silicone polymer) anchored to attachment points (nylon nodes) (17). The strips create force transmission pathways that allow tension to be distributed throughout the suit. The TREINI Exoflex suit is customized to each individual and carried out according to the physical characteristics of each participant. Basic assembly is performed initially; then, adjustments are made to correct the postural imbalances observed in each participant. Basic assembly is performed initially; then, adjustments are made to correct the postural imbalances observed in each participant. The intervention protocol has been subdivided into two phases: (1) myofascial meridian training, lasting 30 min; and (2) functional activity training, lasting 60 min. Both are described in detail in the corresponding sections of this article. All phases of the intervention protocol were carried out at the naturalistic therapeutic environment known as “City of Tomorrow”, at a private clinic, in Ribeirão das Neves, Brazil. It consists of units that simulate real-life contexts, such as home, school, markets, streets, and sport courts (Figure 2). These units provide children with opportunities aimed at engaging in activities relevant to real life, in situations that resemble their natural environment in a playful manner, using concrete materials. The intervention was led by two physiotherapists (RGC and DOS) who have more than a 10-year experience working with children with disabilities in a health care context.

The myofascial meridian training program involved performing activities that induce muscle contractions, engaging all muscle groups associated with each of the different myofascial meridians, including the superficial back line, superficial front line, lateral line, spiral line, and functional line. During training, muscle contraction was performed throughout the range of motion of the myofascial meridians. Thus, the myofascial meridians underwent tension in elongated positions during dynamic activities, promoting capacity for increased elastic energy storage.

**Figure 3:**
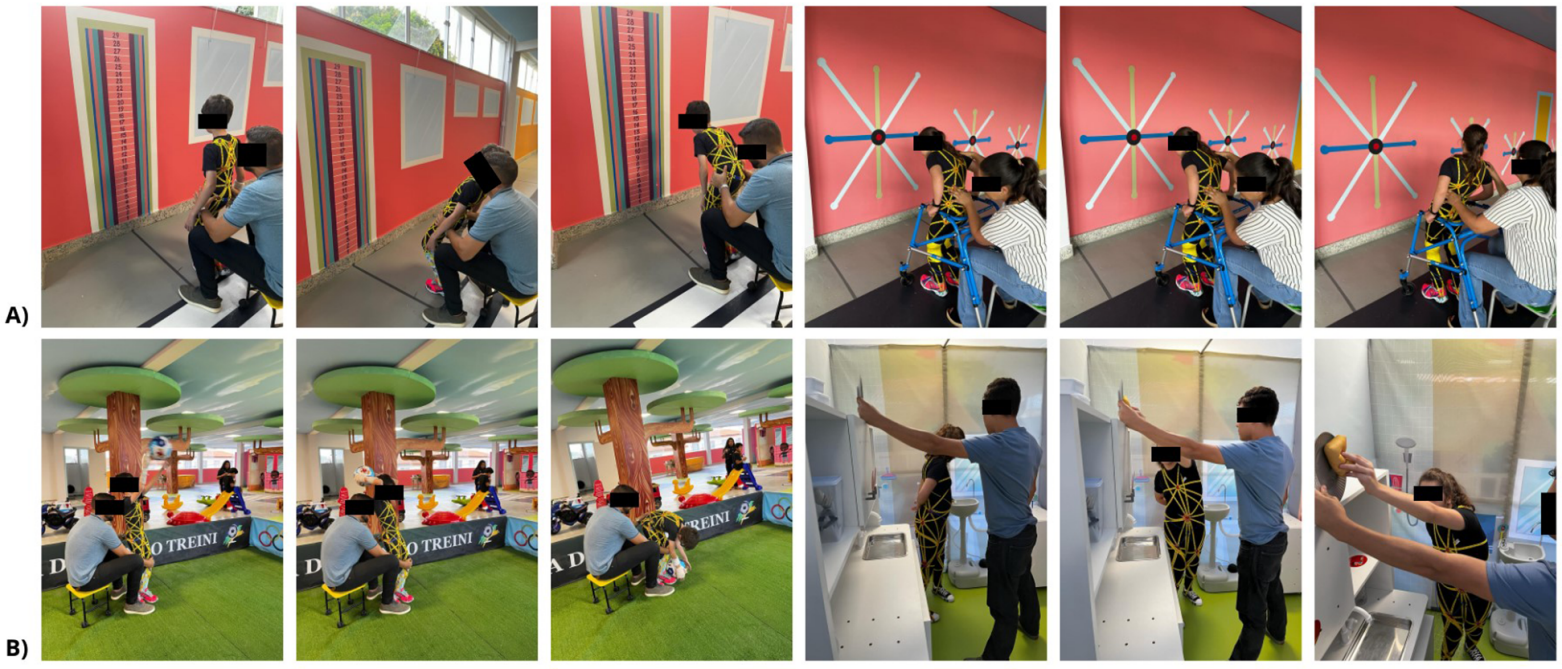
**(A)** Myofascial meridian training programme involving the superficial posterior, superficial anterior, lateral, spiral, and functional lines. **(B)** Training of functional activities involving the myofascial meridians.

To guide each participant in performing the activity, the suit featured a light beam at the level of the sternum manubrium, which was projected onto environmental markings based on the myofascial meridians and were represented previously on panels within the City of Tomorrow. When the participant moved, it caused a change in the direction of the projected light beam attached to the suit. The participant was then instructed to project the light beam onto the environmental markings and follow the outlined path using active and dynamic body movements (Figure 3A). Each movement was repeated between 6 and 10 times, depending on the participant's tolerance. Training was conducted during the initial 30 min of each session.

The functional activities training focused on transferring skills developed in the myofascial meridian exercises to practical, everyday tasks. Some examples include standing from a sitting position, crouching and kneeling, bending and reaching for objects in different planes, throwing, marching, sideways marching, and kicking (Figure 3B). All activities were carried out at the House Unit, Market (“Mercadinho” Unit), and Fitness Space of the City of Tomorrow, using the principles of myofascial meridian training.

### 2.6 Statistical analysis

Measures obtained through PBS and TUG of each child were plotted into individual graphs to allow the visual analysis of the results (31). Graphs were performed on an available website (https://manolov.shinyapps.io/Overlap) suggested by Krasny-Pacini (20). Also, the two-standard deviation (2-SD) band method was used to detect differences between phases. According to this method, two horizontal lines representing two standard deviations above and below the mean data point for the baseline phase were drawn around the data obtained in the baseline and intervention phases (32). Differences between phases were determined when, at least, 2 consecutive data points during the intervention and follow-up phases occurred outside the 2-SD band (32). To provide a measure of treatment effectiveness, the Percentage of Nonoverlapping Data (PND) was computed (33). Specifically, the number of intervention data points that exceed the highest or lowest baseline data point is divided by a total number of intervention data points, and the result is multiplied by 100%. Scruggs and Mastropieri (34, 35) suggest that PND scores above 90 represent a highly effective intervention, scores from 70 to 90 represent effective treatments, scores from 50 to 70 suggest outcomes that are questionable or low, and scores below 50 are ineffective. The Baiobit™ data, PEDI-CAT and COPM scores, were compared between pre- and post-intervention measurements.

## 3 Results

**Figure 4:**
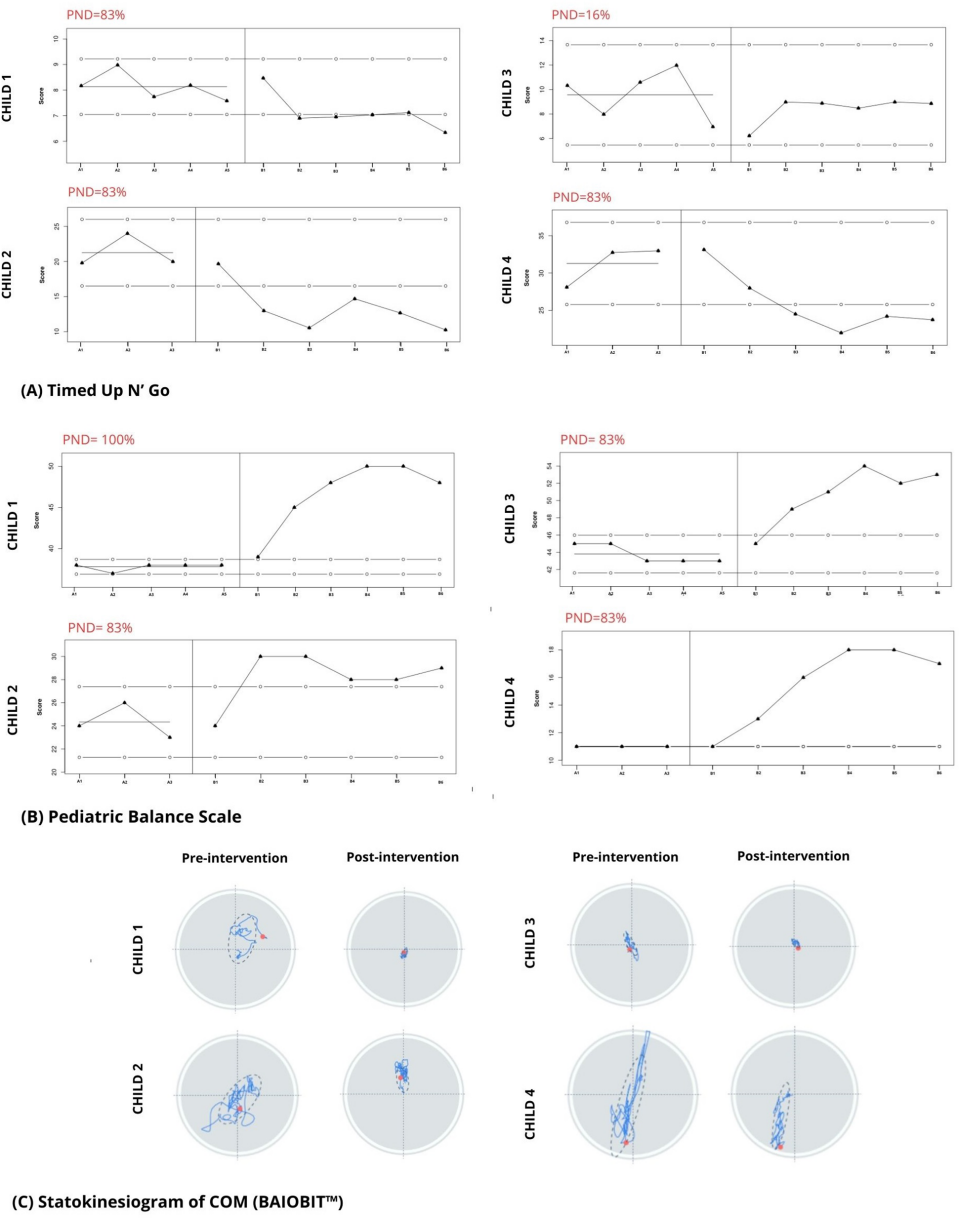
**(A,B)** graphs representing each child during the study. Baseline (A1–A3, A1–A5) and intervention (B1–B6) values are shown; the line marked by solid dots indicates the score at baseline; the lines marked by the open dots indicate the 2-SD band. The percentage of non-overlapping data is shown in red. **(C)** Graphic output of the COM statokinesiogram before and after the intervention.

All four children successfully completed the longitudinal evaluations and the intervention program without the need for any modifications or adjustments, and no adverse events were reported. However, adherence to the program varied slightly among the participants, with adherence rates of 88.89% for child 1, 100% for child 2, 94.44% for child 3, and 77.78% for child 4. The 2-SD band method and PND between the baseline and intervention phases regarding TUG and PBS are shown in Figure 4. Tables 2, 3 show the COP global variables and PEDI-CAT and COPM scores before and after testing. Details of the COPM goals established for each child are shown in the Supplementary Material 1.

**Table 2:** Balance based on COM parameters (Baiobit™).

COM measures	Child 1				Child 2				Child 3				Child 4			
	Open eyes		Closed eyes		Open eyes		Closed eyes		Open eyes		Closed eyes		Open eyes		Closed eyes	
	Pre.	Post.	Pre.	Post.	Pre.	Post.	Pre.	Post.	Pre.	Post.	Pre.	Post.	Pre.	Post.	Pre.	Post.
EA-95% (mm^2^)	634	151	1,894	141	2,512	1,478	3,466	432	116	212	390	99	1,861	1,279	4,123	572
AP-COM (mm)	23	20	44	13	98	70	73	30	18	33	47	15	88	71	191	30
ML-COM (mm)	41	9	53	9	38	40	79	18	6	9	17	8	31	21	46	24
AS-AP (mm/s)	6.17	4.35	7.17	3.99	24.29	16.19	21.32	11.89	4.52	6.13	7.42	3.9	19.6	9.15	33.53	11.58
AS-ML (mm/s)	5.63	2.22	7.71	2.69	9.83	7.95	14.18	6.74	1.26	2.11	3.47	1.69	8.64	5.33	11.2	6.35
TTL (mm)	272	159	346	157	836	589	854	398	147	205	263	136	687	388	1,100	424

Abbreviation: Pre., pre-intervention; Post., post-intervention; EA-95% (mm^2^), ellipse area of 95% confidence; AP-COM (mm), antero-posterior oscillation range of the center of mass (COM); ML-COM (mm), mid-lateral oscillation range of the center of mass (COM); AS-AP (mm/s), average speed of the antero-posterior oscillation of the center of mass (COM); ML-COM (mm), average speed of the mid-lateral oscillation of the center of mass (COM); TTL, total trajectory length of the COM.

**Table 3:** PEDI-CAT and COPM scores.

PEDI-CAT scores	Child 1				Child 2				Child 3				Child 4			
	SS (SD)		T-Score		SS (SD)		T-Score		SS (SD)		T-Score		SS (SD)		T-Score	
	Pre.	Post.	Pre.	Post.	Pre.	Post.	Pre.	Post.	Pre.	Post.	Pre.	Post.	Pre.	Post.	Pre.	Post.
Daily activities	66 (1.39)	63 (0.89)	47	26	57 (0.79)	63 (0.96)	29	48	61 (0.87)	59 (0.7)	31	28	59 (0.79)	62 (1.05)	31	42
mobility	65 (0.72)	65 (0.66)	<10	<10	58 (1.08)	65 (0.76)	<10	17	64 (0.84)	64 (0.6)	<10	<10	57 (1.32)	58 (1.12)	<10	<10
COPM scores	Child 1				Child 2				Child 3				Child 4			
	Performance		Satisfaction		Performance		Satisfaction		Performance		Satisfaction		Performance		Satisfaction	
	Pre.	Post.	Pre.	Post.	Pre.	Post.	Pre.	Post.	Pre.	Post.	Pre.	Post.	Pre.	Post.	Pre.	Post.
Goal 1	7	8	8	10	4	7	2	5	2	4	2	6	1	5	4	5
Goal 2	1	1	1	1	5	8	7	8	3	5	3	7	1	1	1	1
Goal 3	1	1	1	1	7	7	7	7	4	5	4	8	1	5	1	2
Goal 4	1	1	1	1	4	5	4	5	4	5	4	8	3	6	3	2
Goal 5	–	–	–	–	–	–	–	–	5	6	5	8	3	6	3	6

Abbreviation: PEDI-CAT, pediatric evaluation of disability inventory- computer adaptive test; SS, scaled score; SD, standard deviation; T-S, T-Score; COPM, Canadian occupational performance measure; Pre., pre-intervention; Post., post-intervention.

### 3.1 Child 1

Child 1 demonstrated a non-significant increase (2-SD band analysis) in TUG speed, but significant increases in PBS between the initial and intervention phases (Figures 4A,B). The PND scores revealed that the intervention was effective for the TUG measure and highly effective for the PBS measure. In the graphic output of the statokinesiogram, child 1 demonstrated a reduction in COM movements over time (Figure 4C). Table 2 shows an improvement in global COM variables in the eyes-open and eyes-closed conditions. No improvement was observed in the PEDI-CAT score (Table 3). COPM performance and satisfaction scores are shown in Table 3. Two of the four COPM goals were behavioral and did not change with the intervention (Supplementary Material 1). Only one goal was related to balance, which improved one point regarding performance and two points regarding satisfaction.

### 3.2 Child 2

Child 2 demonstrated increased TUG speed and PBS scores during the intervention. PND scores showed that the intervention was effective for both the TUG and PBS measures. The statokinesiogram's graphic output demonstrated a reduction in COM movements over time (Figure 4C). There was improvement for most global COM variables (Table 2). Child 2 showed improvements in both PEDI-CAT domains and in three of the four COPM goals (Table 3).

### 3.3 Child 3

Child 3 did not demonstrate changes in TUG during the intervention; however, PBS scores increased. PND scores revealed that the intervention was effective for the PBS but not the TUG measure (Figure 4). The statokinesiogram's graphic output demonstrated a reduction in COM movements over time. There was a worsening in the global COP variables in the eyes-open condition and an improvement in the eyes-closed condition. There was no improvement in the PEDI-CAT score (Table 3). Child 3 achieved an improvement of at least one point in all COPM goals (Table 3).

### 3.4 Child 4

Child 4 demonstrated improved TUG and PBS scores. The same was observed for the PND. The statokinesiogram's graphic output showed a reduction in COM movements (Figure 4). Table 2 shows an improvement in the global COM variables. Child 4 achieved an improvement in both PEDI-CAT domains as well as improvement in four of the five COPM goals (Table 3).

## 4 Discussion

This study examined the effects of the TREINI Exoflex intervention program on balance and postural control in children with CP. The reach of the intervention in the activity (mobility and ADLs) and participation (goal achievement) outcomes were also evaluated. Using a single-participant, multiple-baseline design, we demonstrated that the intervention improved balance and postural control in all four children. The scope of the intervention regarding activity and participation outcomes varied between the four children. Two children showed improvements in mobility and ADLs. All children showed improvement in at least one goal. Because task-oriented training (36) is widely recommended, it is possible that combining it with the TREINI Exoflex suit could bring greater benefits, especially for goals that require balance. Improvements in goal attainment occurred primarily for balance-related goals, whereas behavioral goals were not achieved.

In this study, PBS and Baiobit scores improved in all children, suggesting direct effects of the TREINI Exoflex suit on balance and postural control. However, our single-participant experimental design (8) suggests that the benefits of the intervention varied between children. Children 1, 2, and 4 showed significant increases (PND >83%) in TUG, which did not happen in child 3. The use of the suit combined with the exercise program may have contributed to improvements in central stability and optimal balance control, factors that impact gait speed (37). The TREINI Exoflex suit can induce activation of the myofascial meridians related to the trunk muscles, facilitating coordination with the limbs and other aspects related to balance, such as activation of sensory receptors, adaptation to the base support, muscle length, and activation of muscle groups. There is evidence that improvements in gait may be linked to trunk muscle activation, which is crucial for monitoring displacement and optimizing steps during walking (38). Furthermore, the postural correction promoted by the garment can affect the ability to take longer steps, which might explain the higher gait speed observed in the TUG. Although these aspects are fundamental to gait, they were not evaluated in this study; therefore, they should be investigated in future research.

According to Camargos et al. (39), therapeutic suits can be good aids in the treatment of children with CP if used correctly and with goals that are compatible with scientific evidence. However, the systematic review by Novak et al. (36) indicated that suit-based therapy does not have any significant additional benefits beyond motor function training. The mechanism of action of therapeutic suits such as the TheraSuit and PediaSuit consists of continuous compression exerted by the elastic elements of the clothing on the child's musculoskeletal system (40). The mechanical model of structural stability has limitations when explaining the stability of body joints, which partly justifies the absence of benefits in the studies already carried out, as pointed out by Novak et al. (36) Bearing in mind these limitations, the mechanism of action of the TREINI Exoflex suit is based on tensegrity. This model is an intrinsically stable system that contains compression components within an interconnected network under continuous tension. Furthermore, the theoretical foundation of the TREINI Exoflex suit is based on the tensegrity model of inherent stability in the musculoskeletal system (16, 17, 41) and was developed to be used during the performance of functional activities, representing the best available evidence in pediatric rehabilitation (17, 36, 42). These characteristics of the TREINI Exoflex suit may explain the benefits associated with using the suit with the participants of this study.

The intervention improved all COP global variables in the closed-eyes condition, demonstrating benefits on proprioception. Myofascial meridians are involved in the organization of movement and proprioceptive functions (4, 13, 14, 41, 43). Studies indicated that fascial sensitivity is greater than joint capsule sensitivity because receptors present in joint capsules are generally stimulated only at extreme joint amplitudes and not during physiological movements, as occurring with fascial proprioceptive receptors (41, 44, 45). As the fascia is rich in proprioceptive receptors and the TREINI Exoflex suit favors its tissue properties, the fascia may become more sensitive to small-joint and multi-joint movements because of reduced fascial adherence promoted by the suit (17, 41).

Improvements in balance and postural control did not necessarily transfer to activity and participation for all four children. Only children 2 and 4 improved regarding mobility and ADLs. Several conclusions can be drawn. First, it is possible that 6 weeks of intervention was not long enough for gains in balance to be transferred to mobility tasks and ADLs, which were not directly targeted by the intervention. Furthermore, there was no linear relationship between International Classification of Functioning, Disability and Health domains; therefore, gains in balance will not necessarily result in improvements in ADLs or mobility. Second, all four children performed below average on the intelligence tests. Previous research showed that children with CP with intellectual disabilities have lower performance thresholds in ADLs earlier than children without disability (46). It is possible that the cognitive abilities of the participants have influenced their progress in ADLs. However, this remains speculative and warrants further investigation into future studies. The third observation concerns the lack of improvement in mobility and the decline in performance in ADLs observed in children 1 and 3, who were 14 and 12 years old, respectively. These ages are significant given that previous research suggests that, for certain categories of PEDI-CAT ADLs, children generally reach 90% of their performance potential between the ages of 12 and 14 (46). This developmental plateau may potentially be responsible for the less favorable outcomes observed in these older children. However, it is important to note that this is a hypothesis based on existing evidence, and further studies are needed to comprehensively understand the effects of this intervention on ADLs and mobility across different age groups. For child 1, regarding the four goals in the COPM, only one related to balance showed improved performance and satisfaction scores. A similar result was observed for child 4, who improved in three of the four balance-related goals. In addition to reinforcing the benefits of the intervention regarding balance, these results draw attention to the importance of the specificity of the intervention for achieving goals. No behavioral intervention was used in this study, which justifies the lack of improvements in most of the goals established for child 1.

This study has several limitations that warrant consideration. The small sample size of only four participants limits the generalizability of the findings. The absence of long-term follow-up after the intervention further limits our understanding of the durability of the observed improvements. Therefore, follow-up studies are recommended to assess the persistence of the intervention effects over extended periods. Future research should also consider including a broader range of participants across GMFCS levels, including individuals from levels IV and V of the GMFCS, and extending the duration of the intervention to validate the findings better. While the methods used are robust and appropriate for the small sample size, incorporating statistical analyses like 2-SD banding and percentage of non-overlapping data (PND), the limited number of baseline assessments and lack of blinding could introduce bias. Follow-up studies should aim to address these limitations by employing larger sample sizes, longer intervention and follow-up periods, and possibly a blinded design to enhance the reliability and applicability of the results.

## 5 Conclusion

This is the first study to assess the benefits of an intervention program utilizing the TREINI Exoflex suit for children with CP. The findings from this study suggest that the TREINI Exoflex intervention program can significantly enhance balance and postural control in children with CP, indicating its potential for integration into routine clinical practice. The findings of this initial study contribute to advances in the rehabilitation of children with CP. By employing a structured, individualized approach, the program effectively addressed specific balance-related goals across all participants, demonstrating the suit's utility in tailored therapeutic interventions. The variation in improvements regarding mobility and ADLs among the children highlights the need for personalized assessment and implementation strategies within clinical settings. Integrating TREINI Exoflex into routine practice could provide clinicians with a robust tool to enhance traditional rehabilitation methods, offering a complementary modality that addresses both functional mobility and individualized goal attainment. Furthermore, the adherence rates, although variable, suggest that the program is well-tolerated, indicating that with appropriate motivational strategies and family support, adherence could be optimized. The documented improvements in participation outcomes underscore the suit's potential to enhance the quality of life for children with CP, making it a valuable asset in the clinical management and long-term therapeutic planning for this population. As such, the TREINI Exoflex suit could be incorporated as part of a comprehensive rehabilitation program, fostering a holistic approach to pediatric CP management by enhancing motor outcomes and supporting goal-oriented therapy. Additionally, exploring the program's implementation in diverse settings beyond the controlled study environment could offer further understanding of its practical applicability and effectiveness. This could include adaptations for various clinical and community contexts, ensuring that the intervention is both accessible and effective for a wider range of children with cerebral palsy. Future studies should aim to address these aspects, thereby enhancing the generalizability of the findings and supporting the integration of the intervention into routine clinical practice for a more inclusive population. Also, in future research, conducting randomized controlled trials and follow-up studies could provide more convincing results on the effectiveness of TREINI Exoflex and evaluate the long-term persistence of its intervention effects.

## Data Availability

The raw data supporting the conclusions of this article will be made available by the authors, without undue reservation.
