# Supplementary material for: Effects of a therapeutic suit based on myofascial meridians on postural control and balance in children with cerebral palsy: a multiple-baseline, single-subject study
Source: Front Pediatr. 2025 Jan 20;13:1459839. doi: 10.3389/fped.2025.1459839 (PMC11788328; doi:10.3389/fped.2025.1459839)
Supplement: Supplementary file 1 [file Table1.docx]

Supplementary Material 1

# Supplementary Tables: COPM goals established for each child

|  | | | **Performance** | | **Satisfaction** | |
| --- | --- | --- | --- | --- | --- | --- |
| **Child** | **Goal** | **Importance** | **Pre** | **Pro** | **Pre** | **Pos** |
| **1** | Static balance (need support) | 10 | 7 | 8 | 8 | 10 |
|  | (Doesn't do homework) (behavior) | 10 | 1 | 1 | 1 | 1 |
|  | (Doesn't do school activities (behavior) | 10 | 1 | 1 | 1 | 1 |
|  | Tie shoelace | 10 | 1 | 1 | 1 | 1 |
| **2** | Cut meat | 8 | 4 | 7 | 5 | 5 |
|  | Soaping your back | 6 | 5 | 8 | 7 | 8 |
|  | Walking on the street (obstacles) | 8 | 7 | 7 | 7 | 7 |
|  | Tie shoelace | 8 | 4 | 5 | 4 | 5 |
| **3** | Wearing clothes that are inside out | 9 | 2 | 4 | 2 | 6 |
|  | Wash private parts (take a shower standing up) | 9 | 3 | 5 | 3 | 7 |
|  | Wash private parts (take a shower standing up) | 9 | 4 | 5 | 4 | 8 |
|  | Articulate words better (when speaking) | 9 | 5 | 5 | 5 | 8 |
|  | Write the name without looking | 9 | 5 | 6 | 5 | 8 |
| **4** | Cut meat | 10 | 1 | 5 | 4 | 5 |
|  | Stand without support (balance) | 10 | 1 | 1 | 1 | 1 |
|  | Walking with support (holding one hand) (legs crossed) | 10 | 1 | 5 | 1 | 2 |
|  | Open base to get up from a chair | 10 | 3 | 6 | 3 | 2 |
|  | Moves to sit on a chair/sofa | 10 | 3 | 6 | 3 | 6 |
